# High-Thermal-Conductivity Graphene/Epoxy Resin Composites: A Review of Reinforcement Mechanisms, Structural Regulation and Application Challenges

**DOI:** 10.3390/polym17172342

**Published:** 2025-08-28

**Authors:** Hongwei Yang, Zongyi Deng, Minxian Shi, Zhixiong Huang

**Affiliations:** 1 School of Materials Science and Engineering, Wuhan University of Technology, Wuhan 430070, China; 2 Hubei Longzhong Laboratory, Xiangyang 441000, China

**Keywords:** graphene, epoxy resin, composite materials, thermal conductivity, applications

## Abstract

As electronic devices advance toward higher power density, heat dissipation has emerged as a critical bottleneck limiting their reliability. Graphene oxide (GO)/epoxy resin (EP) composites, combining high-thermal-conductivity potential with polymer-matrix advantages, have become a key focus for overcoming the limitations of traditional metal heat-dissipation materials. This review systematically examines these composites, analyzing their thermal conductivity enhancement mechanisms, structural regulation strategies, and application challenges. We first elaborate on how GO’s intrinsic properties influence its enhancement capability, then explore the roles of physical dispersion strategies and interfacial modification techniques in optimizing filler dispersion and reducing interfacial thermal resistance, revealing the effects of preparation processes on thermal conduction network construction. Their remarkable potential is demonstrated in applications such as electronic packaging and electromagnetic shielding. However, challenges including cross-scale structural design and multi-physics collaborative regulation remain. This review aims to provide theoretical foundations and technical guidance for transitioning these composites from lab research to industrial application and advancing thermal management in high-performance electronics.

## 1. Introduction

Amid rapid technological advancement, high-thermal-conductivity materials are critical in electronics, aerospace, and new energy [1,2,3,4,5]. Advancing microelectronics toward high performance, miniaturization, and integration has intensified operational heat generation, making dissipation a key bottleneck for device reliability [2,6,7]. Traditional metal-based materials (e.g., copper, aluminum) increasingly fail to meet demands due to high density, mismatched thermal expansion, and limited processability [8,9,10]. Consequently, graphene oxide (GO)/epoxy resin (EP) composites, with their unique physicochemical structures and high-thermal-conductivity potential, have emerged as a research focus.

Polymer-matrix composites offer potential due to low density, processability, and corrosion resistance. EP, a key thermosetting matrix, is widely used for its excellent adhesion, electrical insulation, and mechanical properties [1,10]. However, EP’s inherently low thermal conductivity restricts its thermal management applications. Enhancing polymer conductivity relies on high-thermal-conductivity fillers [9,11,12]. Among nanofillers, GO stands out with its 2D honeycomb lattice, theoretical 5000 W·m^−1^·K^−1^ intrinsic thermal conductivity, and mechanical properties, making it ideal for high-thermal-conductivity composites [13,14]. Achieving performance leaps in GO/EP composites via structural design and process optimization remains a core challenge.

**Figure 1:**
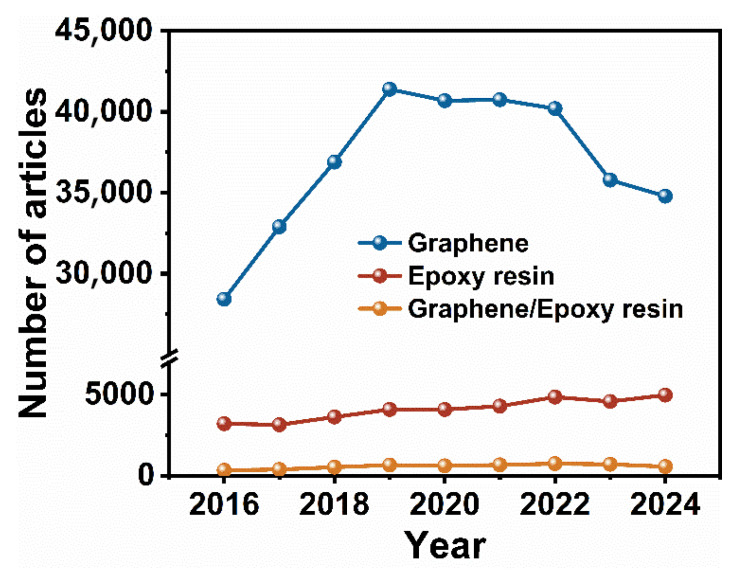
Based on the number of papers published annually in the Web of Science database.

Web of Science analysis indicates significant growth in GO/EP research (Figure 1). Scholars have elucidated GO’s heat-transfer mechanisms in composites and proposed multi-scale enhancement theories (e.g., phonon coupling, interfacial dissipation, and network topology optimization) [15,16]. Efficient fabrication, however, faces critical challenges: van der Waals forces cause GO agglomeration, leading to uneven dispersion and poor thermal network connectivity [13,17]; poor GO-EP interfacial compatibility induces high Kapitza resistance, impairing heat transfer [1,6,18]; and composite conductivity depends on GO orientation, 3D-network topology, and filler-matrix interactions, requiring precise regulation [19]. Large-scale production further complicates these issues due to process costs, structural stability, and performance optimization [20]. Addressing these demands requires multidimensional, cross-scale research on filler control, interface engineering, and advanced preparation methods.

This review centers on the “structure-performance-application” framework, systematically analyzing the reinforcement mechanisms, structural regulation strategies, and application challenges of high-thermal-conductivity GO/EP composites to establish a scientific analysis framework. It examines graphene’s intrinsic properties, explores how dispersion methods and interface modification affect filler distribution and thermal resistance, and investigates thermal network construction via solution mixing and in situ polymerization. Application bottlenecks and optimization strategies in electronic packaging and electromagnetic shielding are highlighted. Through multidimensional analysis, it reveals microstructure-macroscopic performance relationships, clarifies current limitations, outlines future directions, and provides theoretical and technical support for high-performance composite design.

## 2. Basic Properties of Graphene

Wallace pioneered the electronic structure framework in 1947. In 2004, Geim’s team prepared and observed the quantum Hall effect via micromechanical exfoliation, overcoming Mermin-Wagner constraints and bridging the carbon dimensional hierarchy gap (0D fullerene to 3D diamond) [21,22]. Its distinctive carrier transport, mechanical strength, and optical properties render it ideal for novel composites, flexible electronics, and related fields.

### 2.1. Basic Structure of Graphene

**Figure 2:**
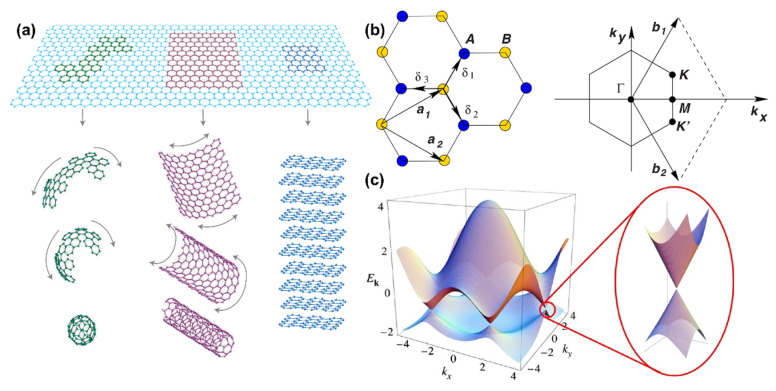
(**a**) Relationship between GO and other carbon materials [33]. Copyright 2007, Springer Nature; (**b**) Honeycomb lattice of GO and its Brillouin zone; (**c**) Band structure of GO [23]. Copyright 2009, American Physical Society.

Figure 2a shows GO, which is a fundamental building block among carbon allotropes that include zero-dimensional fullerenes, one-dimensional carbon nanotubes, and three-dimensional graphite. This structure has a sub-nanometer thickness and demonstrates both Dirac-cone band characteristics and zero-bandgap semiconductor properties, providing the theoretical basis for its remarkable physical performance [21,22].

According to the tight-binding approximation theory, the honeycomb lattice of graphene consists of a unit cell composed of two atoms. The basis vectors in real space are denoted as $a_{1}=\frac{a}{2}\left(3,\sqrt{3}\right)$ and $a_{2}=\frac{a}{2}\left(3,-\sqrt{3}\right)$ and the nearest neighbor atomic position vectors ***δ***_1,2,3_ satisfy sixfold symmetry [23,24] (Figure 2b). The basis vectors of the reciprocal space are $b_{1}=\frac{2\pi }{3a}\left(1,\sqrt{3}\right)$ and $b_{2}=\frac{2\pi }{3a}\left(1,-\sqrt{3}\right)$ and Dirac cones are present at the vertices $K=\left[\frac{2\pi }{3a},\frac{2\pi }{3\sqrt{3}a}\right]$ and $Kʹ=\left[\frac{2\pi }{3a},-\frac{2\pi }{3\sqrt{3}a}\right]$ of the Brillouin zone [23,24,25]. Transitions between nearest neighbors (t ≈ 2.8 eV) and second-nearest neighbors (t′ ≈ 0.1 t) are taken into account by the tight-binding model Hamiltonian [24], which has the dispersion relation $E_{\pm }\left(k\right)=\pm t\sqrt{3+f\left(k\right)}-t^{\prime }f\left(k\right)$, where $f\left(k\right)=2cos\left(\sqrt{3}k_{y}a\right)+4cos\left(\frac{\sqrt{3}}{2}k_{y}a\right)cos\left(\frac{3}{2}k_{x}a\right)$. As seen in Figure 2c, massless Dirac fermion behavior is revealed by the linear dispersion $E_{\pm }\left(q\right)\approx \pm v_{F}\left|q\right|+O\left[{\left(\frac{q}{K}\right)}^{2}\right]$ expanded at k = K + q ($\left|q\right|\ll \left|K\right|$) and the Fermi velocity vF ≈ 106 m/s [26,27,28].

Given GO’s 2D quantum properties and heterostructure, comprehensive characterization requires complementary techniques. Scanning tunneling microscopy (STM) enables atomic-scale morphology analysis, revealing the graphene honeycomb lattice and electronic state distribution [29]. Transmission electron microscopy (TEM) directly visualizes hexagonal ring atomic arrangement via electron-scattering imaging; combined with selected-area diffraction, it verifies reciprocal-space structure [30]. Angle-resolved photoemission spectroscopy (ARPES) quantifies Dirac-cone energy band linear dispersion, validating massless Dirac fermion behavior [31,32].

### 2.2. Basic Performance Characteristics of Graphene

GO, a multifunctional material with a 2D honeycomb lattice (specific surface area: 2630 m^2^·g^−1^), exhibits exceptional mechanical strength (theoretical Young’s modulus: 1.0 TPa), electrical properties (room-temperature carrier mobility > 15,000 cm^2^·V^−1^·s^−1^), and thermal conductivity (single-layer: 5000 W·m^−1^·K^−1^) [13,17,34]. As shown in Table 1. However, its zero-bandgap nature limits electronic applications. Bandgap opening via lattice/chemical disruption (e.g., doping, hydrogenation) or non-destructive modulation (electric field, stress, substrate coupling) struggles to maintain high mobility and large-area preparation [35,36].

Electrical properties arise from its Dirac-cone band structure, where conduction and valence bands linearly cross at Brillouin zone K/K’ points, endowing carriers with relativistic transport and enabling the room-temperature quantum Hall effect; bandgapization requires symmetry breaking [23,27]. Optical features include broad-spectrum constant absorption, high transmittance, nonlinear response (adjustable third-order polarizability), low loss, and electrically programmable surface-isotropic excitations, suiting optoelectronics [25,37,38]. Mechanical properties originate from sp^2^ covalent networks but are influenced by interlayer van der Waals forces, defects, and temperature [39,40]. Thermal transport relies on low-dimensional phonon transport (acoustic branch dominated); practical applications require reducing interfacial thermal resistance via functionalization [17,41,42]. Its diverse applications include fuel cells and environmental adsorption (chemical stability/conductivity) [43,44], spintronics (ferromagnetic modification) [45], and medication delivery/biosensing (biocompatibility/antibacterial properties) [46,47].

**Table 1:** Properties of graphene [21,48,49]. Copyright 2021, Elsevier. Copyright 2024, Elsevier. Copyright 2021, Elsevier.

Property	Value
Density/(g·cm^−3^)	2.3
Thermal conductivity/(W·m^−1^·K^−1^)	5000
Youngʹs modulus/TPa	1.0
Fracture strength/GPa	130
Tensile strength/GPa	100
Shear modulus/GPa	280
Longitudinal sound velocity/(km·s^−1^)	20
Current density (A·cm^−2^)	2 × 10^9^
Melting temperature/K	4900
Specific surface area/(m^2^·g^−1^)	2630
Light transmittance/%	97.70
Carrier mobility/(cm^2^·v^−1^s^−1^)	2.5 × 10^5^
Interlayer spacing/nm	0.335

### 2.3. Preparation of Graphene

**Figure 3:**
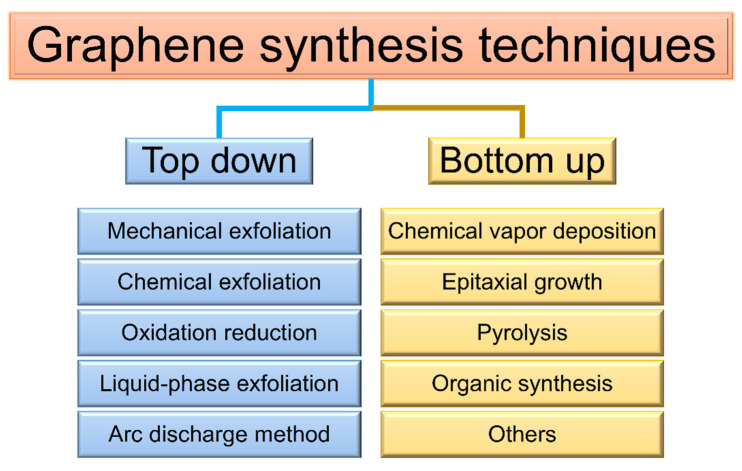
Methods for preparing graphene.

In 2004, Geim and Novoselov successfully prepared stable GO via tape exfoliation, inaugurating the era of two-dimensional material research [50]. Generally, GO preparation methods are classified into top-down and bottom-up approaches [22] (Figure 3). Mechanical exfoliation, redox methods, chemical vapor deposition (CVD), and other common techniques exhibit limitations in yield, defect control, and scalability. Mechanical exfoliation disrupts van der Waals interactions between graphite layers (bond energy: ~2 eV·nm^−2^, interlayer spacing: 3.34 Å), enabling precise fabrication of high-quality single- or few-layer graphene [33,50]. Despite low yield and small area, it remains a valuable method for van der Waals heterojunction and quantum transport research [22,34]. The redox method, based on Hummer’s process, uses strong oxidation to generate GO with oxygen-functional groups, followed by chemical or thermal reduction to restore the sp^2^ network and produce reduced graphene oxide (RGO) [22,51]. Chemical reduction enhances conductivity but generates hazardous residues, whereas thermal reduction enables kilogram-scale continuous production but induces structural damage. CVD, the most widely used industrial technique, enables epitaxial growth via catalytic decomposition of carbon sources. Copper substrates have emerged as the preferred choice for industrialization, attributed to their single-layer preparation capability and low cost. However, surface nanosteps on copper substrates promote lateral growth, and their flatness directly influences film quality [35,52]. To mitigate transfer damage, transfer-free technologies (e.g., PECVD), liquid metal substrates, and alloy substrates have been developed, while roll-to-roll process optimization has enhanced wafer compatibility and cost-effectiveness [53,54,55,56].

## 3. Improve the Dispersibility and Interface Compatibility of Graphene

Material macroscopic properties depend on surface characteristics, where atomic configuration, electronic structure, and chemical state differences dominate physical and chemical behavior. For polymer composites, the core challenge is optimizing filler dispersion and interface compatibility to construct efficient heat conduction networks. GO, with its sp^2^-bonded 2D structure and excellent properties, shows great potential, but its high specific surface area and strong van der Waals forces cause agglomeration, inducing defects and reducing enhancement effects. While existing dispersion strategies mitigate π-π stacking and high exfoliation energy barriers, inherent interface incompatibility between polymer matrices and graphene leads to weak chemical adhesion, resulting in strong phonon scattering and high Kapitza thermal resistance during heat transfer, severely limiting efficiency [15,34]. Thus, precise interface performance control is critical for high-performance graphene/polymer composites.

### 3.1. Physical Dispersion Method

The physical dispersion method disrupts van der Waals forces and π-π interactions between graphene layers via external forces, achieving uniform dispersion in polymer matrices. It has gained popularity due to its simplicity and rapidity.

**Figure 4:**
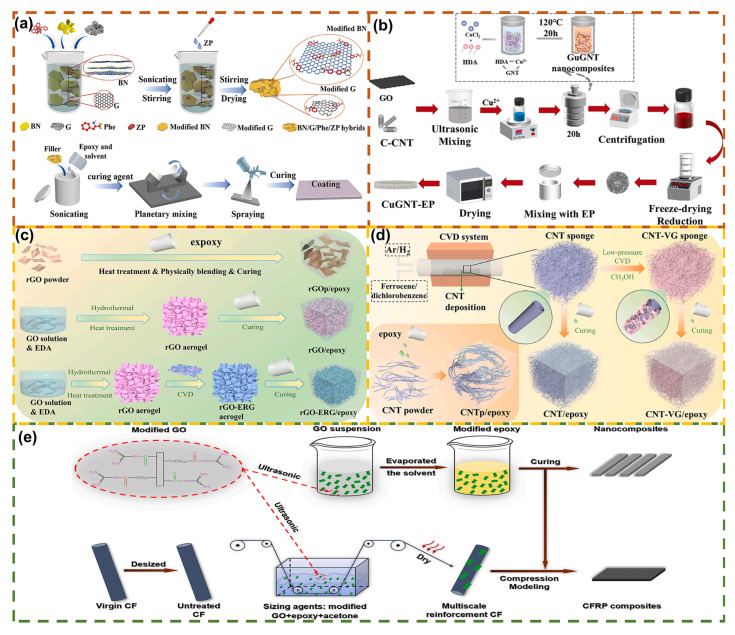
(**a**) Schematic diagram of the preparation of BN/G/Phe/ZP and BN/G/Phe/ZP/EP coatings [60]. Copyright 2022, Elsevier; (**b**) schematic diagram of the preparation of CuGNT-EP coatings [73]. Copyright 2025, Elsevier; (**c**) schematic diagram of the preparation of RGO-ERG/epoxy composites [80]. Copyright 2022, Elsevier; (**d**) Schematic diagram of the preparation of three types of epoxy resin composites (CNTp/epoxy, CNT/epoxy and CNT-VG/epoxy) [86]. Copyright 2023, Elsevier; (**e**) schematic diagram of the preparation of CFRP/EP composites [93]. Copyright 2021, Elsevier.

Ultrasonic processing exfoliates and disperses graphene through the cavitation effect, where efficiency depends on power, frequency, duration, and solvent [57]. High power enhances dispersion but may induce structural damage, while low frequency improves cavitation but elevates the risk of layer fracture [58]. Polar solvents (e.g., NMP, DMF) enhance cavitation, while aqueous/surfactant systems meet biocompatibility requirements [58,59]. Xu et al. [60] prepared a composite coating with a thermal conductivity of 1.63 W·m^−1^·K^−1^ (Figure 4a), reducing the heat-transfer coefficient loss of the heat exchanger to 1.39%. Lu et al. [61] produced GO nanosheets (lateral dimension: 1.8 μm, thickness: 1.5 nm) to fabricate a composite with 262 W·m^−1^·K^−1^ thermal conductivity, enhancing heat-dissipation efficiency by 68.2%. Ma et al. [62] developed GO/EP composites with high thermal conductivity (6.81 W·m^−1^·K^−1^) and excellent microwave absorption (minimum reflection loss: −24.15 dB). However, this method exhibits drawbacks: limited yield, challenges in layer size control, increased defects from prolonged ultrasonication, and uneven cavitation dispersion [63,64].

Ball milling exfoliates GO via shear/collision stress fields from grinding media, applicable in dry or wet modes [34]. The wet mode uses solvents and surfactants to regulate interfacial energy and prevent re-agglomeration [34,65]. Key parameters require coordinated tuning. Chen et al. [66] prepared a water-based dispersion via 12 h ball milling, yielding UL-94 V-0 rated EP composites (limiting oxygen index: 30.5%). Zhang et al. [67] fabricated Cu-MOF-coated graphene flame retardants, reducing EP’s PHRR by 55%. Meng et al. [68] ball-milled in situ modified GO, increasing EP’s elastic modulus by 889%. Ba et al. [69] constructed a 3D graphene network, yielding a composite (4.14 wt% loading) with 35.23 dB electromagnetic interference and 1.19 W·m^−1^·K^−1^ thermal conductivity.

Mechanical stirring achieves separation and dispersion by disrupting interlayer van der Waals forces through fluid dynamics, involving a three-way balance of reduced wetting energy, deagglomeration, and adsorption stability [49,70]. Stirring intensity must be maintained within the critical Reynolds number range: insufficient intensity reduces separation efficiency, while excessive intensity causes platelet contraction [71]. Paddle-type stirrers are optimal for low-viscosity fluids, whereas turbine-type stirrers enhance shear in high-viscosity fluids [72]. Yuan et al. [73] prepared a composite with 0.356 W·m^−1^·K^−1^ thermal conductivity (Figure 4b). Bandeira de Souza et al. [74] demonstrated that functionalized GO enhances EP cure efficacy. Li et al. [75] developed a composite coating with 5.65 W·m^−1^·K^−1^ thermal conductivity. However, this method exhibits poor dispersion, susceptibility to agglomeration, and rapid structural damage from high shear strength [76].

### 3.2. Chemical Modification Method

Chemical modification enhances GO dispersibility in solvents or matrices by modifying surface functional groups (hydrophilic/organophilic) [77,78]. GO is synthesized via graphite oxidation using Hummer’s process, yielding high-density oxygen-containing functional groups on its surface/edges [79]. Following ultrasonic/mechanical exfoliation, single-layer GO is obtained, then partially restored to an sp^2^ conjugated structure via chemical/thermal reduction (retaining functional groups to preserve dispersibility) [78]. Han et al. [80] fabricated 3D epoxy composites (Figure 4c) with 45.9 dB X-band electromagnetic shielding and 1.96 W·m^−1^·K^−1^ thermal conductivity. Hong et al. [81] utilized 3D-printed EP composites, where flake orientation enhanced thermal conductivity and accelerated the shape memory process. Xie et al. [82] developed 5G packaging materials (microwave absorption: −48.28 dB @ 5 GHz; thermal conductivity: 1.6 W·m^−1^·K^−1^). Cui et al. [83] treated 3D graphene aerogel with titanate, yielding a 1388% increase in EP thermal conductivity at 2.5 wt% loading. However, intense oxidation rapidly induces defects, leaving residual oxygen in RGO, and the oxidation process generates wastewater [84,85]. The inherent hydrophobicity of GO restricts its applications, with surface functionalization (covalent/non-covalent) serving as a critical enhancement strategy [52]. Han et al. [86] constructed a 3D hybrid carbon framework (Figure 4d) with 46.9 dB electromagnetic shielding, 2.23 W·m^−1^·K^−1^ thermal conductivity, and a 349.39% enhancement in EP composites’ bending strength. Yan et al. [87] loaded mesoporous SiO_2_ with MBT to enhance GO dispersion and achieve pH-responsive corrosion protection. Wang et al. [88] fabricated a functionalized graphene/SiCnw composite framework with 5 wt% SiCnw, yielding 1.58/6.20 W·m^−1^·K^−1^ thermal conductivity.

To optimize interfacial properties, GO necessitates dispersants (e.g., surfactants or graft polymers). Xu et al. [89] utilized Py-PEG-Py to disperse GO/rGO: 0.03 wt% GO combined with 3.0 wt% dispersant yielded an EP bending strength of 113.2 MPa (56.5% increase). Chen et al. [90] optimized the GO/EP method, yielding a 129% enhancement in thermal conductivity. Thieu et al. [91] utilized cryogenic casting to construct vertical GF scaffolds; 15 vol% GF achieved 0.96 W·m^−1^·K^−1^ through-plane thermal conductivity, representing a 465% increase.

In situ polymerization constructs a dispersed system by interacting monomers with GO surface functional groups, preventing agglomeration during polymerization and enhancing interfacial stress transfer [22,92]. Zhang et al. [93] achieved 30.3% and 32.6% enhancements in tensile strength and interlaminar shear strength, respectively, via carbonyl-grafted polyetheramine (Figure 4e). Zhou et al. [94] prepared a GO-based waterborne epoxy curing agent, enhancing the coating’s mechanical properties and corrosion resistance. Yang et al. [95] fabricated a solvent-free epoxy resin via in situ SiO_2_ deposition; 0.8 wt% nanoparticles increased flexural strength by 24.6% to 39.2 MPa.

In summary, physical dispersion disrupts van der Waals forces via mechanical exfoliation, preserving graphene’s essential structural features but suffering from drawbacks including low dispersion concentration and susceptibility to agglomeration. Chemical dispersion enhances solvent compatibility and stability via modification, yielding stronger interfacial interactions, yet may degrade the structure and introduce defects or impurities.

## 4. Thermal Conductivity Mechanism and Preparation Process of Graphene/Epoxy Composite Materials

### 4.1. Mechanism of Enhanced Thermal Conductivity

#### 4.1.1. Heat-Transfer Mechanism

**Figure 5:**
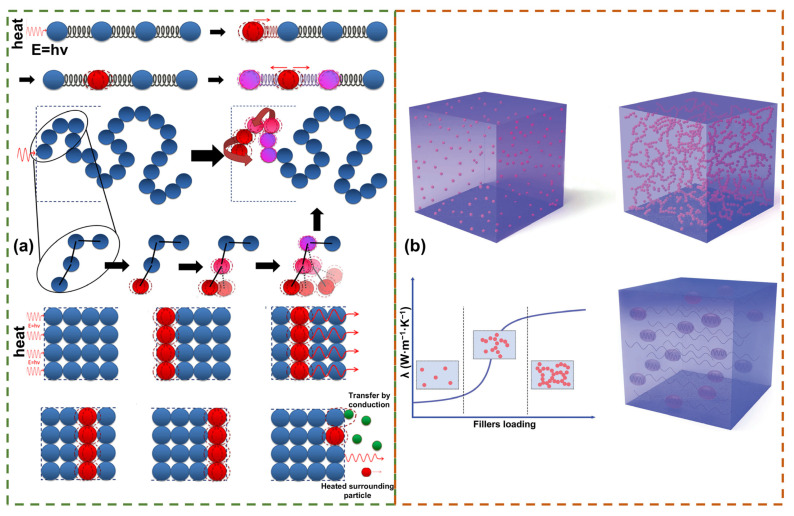
(**a**) Schematic diagram of heat conduction and conduction mechanisms in amorphous and crystalline materials [96]. Copyright 2016, Elsevier. (**b**) Thermal conductivity mechanism of low/high filler composites [97]. Copyright 2023, John Wiley and Sons.

The second law of thermodynamics describes spontaneous heat transfer from high to low temperatures, rooted in temperature gradient-driven disordered motion and energy exchange in microscopic particles [96] (Figure 5a). Non-metallic solids (e.g., polymers, ceramics) transfer heat via lattice vibrations (phonons); however, lattice defects and disordered structures scatter phonons, reducing thermal conductivity [15,97]. Liquids and gases primarily transfer heat via molecular collisions, with lower thermal conductivity than solids. Natural heat transfer occurs via three modes—conduction, convection, and radiation—with conduction dominating in solids and strongly linked to material microscopic structure and particle dynamics [97]. Figure 5b shows the thermal conductivity mechanism of composite materials with low/high fillers.

Heat conduction is heat transfer independent of macroscopic material displacement, occurring within objects or at contact interfaces. Its microscopic mechanism arises from the thermal motion of molecules, atoms, and free electrons [15]. Building on prior work, Joseph Fourier formulated Equation (1) [96], stating that heat flux density is proportional to the temperature gradient perpendicular to the heat-transfer cross-section.

(1) $$q=-\frac{\Phi }{A}=-\lambda \frac{\partial t}{\partial x}$$

Here, *Φ* denotes heat flow—the total heat passing through area *A*. As the temperature gradient is a vector, heat flux density is also a vector, directed along the temperature decrease (opposite to the gradient). This derivation yields the mathematical expression of Fourier’s law, given by Equation (2) [15,96]:

(2) $$q=-\lambda gradt=\lambda \frac{\partial t}{\partial n}n=-\lambda \left(\frac{\partial t}{\partial x}i+\frac{\partial t}{\partial y}j+n\frac{\partial t}{\partial z}k\right)$$

Thermal disturbances within an object induce temperature changes, altering the temperature distribution and heat flux density throughout the object. Under these conditions, Fourier’s law is modified to Equation (3) [15]:

(3) $$\frac{a}{c^{2}}\frac{\partial q}{\partial \tau }+q=-\lambda gradt$$

In the equation, *α* denotes the material’s thermal diffusivity, *c* the thermal propagation velocity, and *α/c^2^* is defined as the relaxation time.

The proportionality constant λ in Fourier’s law, known as thermal conductivity, characterizes a material’s heat-transfer capability. A higher λ indicates a stronger heat-transfer ability [96]. A material’s heat-transfer mechanism involves collisions and energy transfer between its internal microscopic particles, essentially a process where high-amplitude atoms/molecules excite low-amplitude ones to vibrate [96,97].

#### 4.1.2. Heat-Transfer Mechanism of Graphene

GO exhibits extremely high intrinsic thermal conductivity due to its two-dimensional honeycomb lattice, a property driven by phonon-dominated ballistic transport. Its continuous hexagonal conjugated structure suppresses grain boundary scattering, while strong covalent bonds between lightweight carbon atoms yield phonon group velocities near the speed of sound [24,97,98]. GO shows marked thermal anisotropy: in-plane λ is extremely high, but interlayer axial λ drops sharply due to van der Waals forces. Key factors include crystal defects (e.g., vacancies and Stone-Wales dislocations, which reduce phonon mean free path via enhanced scattering) and dimensional effects (increased layers or reduced lateral dimensions causing nonlinear λ decay) [15,99]. Thermal conductivity generally depends on filler dispersion, intrinsic λ, and network topology. While nanofillers form continuous thermal networks for low-scattering transport, amorphous polymer chains induce strong phonon-interface scattering, causing measured λ in graphene/polymer nanocomposites to be orders of magnitude lower than theoretical values, underscoring the phonon-interface transport bottleneck [96,100].

#### 4.1.3. Heat-Transfer Mechanism of Epoxy Resin

EP’s intrinsic thermal conductivity is typically limited to 0.17–0.21 W·m^−1^·K^−1^, a bottleneck arising from triple molecular-scale confinement effects [15,16,19,97,101,102]. First, its saturated molecular structure lacks free electron heat pathways. Second, disordered molecular chains in amorphous EP restrict atomic thermal vibration freedom. Third, phonons (lattice vibration quanta), the primary thermal carriers, diffuse only via localized, stepwise atomic vibrations, causing significant energy dissipation. To address this, intrinsic thermally conductive liquid crystal EP forms ordered crystalline-like orientations in its cross-linked network. Regular intercrystalline lattices enable efficient phonon propagation along orientation directions, while covalently bonded intercrystalline frameworks suppress phonon scattering. Limited by polymer dynamics, it forms a “mesocrystalline-amorphous” two-phase structure, yet maintains macroscopic isotropy due to multi-directional mesocrystalline distribution [102,103]. This micro-nano ordering enhances the phonon mean free path, increasing λ.

However, polymer thermal conductivity remains fundamentally constrained at the molecular chain level. High molecular weight and broadly distributed chain segments restrict thermal vibrations to disordered atomic rotations, requiring phonon transport to transition across heat sources, surface atoms, and adjacent chains [104,105]. Additionally, four structural defects (point, surface, volume, and topological) synergistically induce phonon scattering, limiting λ to ~0.2 W·m^−1^·K^−1^ (Table 2). The interplay of restricted molecular motion and defect scattering defines the theoretical upper limit of polymer thermal conductivity.

#### 4.1.4. Heat-Transfer Mechanism of Composite Materials

Enhancing thermal conductivity in polymer composites relies on constructing thermally conductive filler networks, explained by three synergistic theories [15,16,19,24,97]. (1) Thermal conduction path theory, the dominant framework, describes three network evolution stages: Below the percolation threshold (φ < φ_c_), fillers form discrete “sea-island” distributions, with matrix-dominated phonon scattering limiting λ increase; at φ ≈ φ_c_, fillers contact to form conduction chains; and above φ_c_, a 3D continuous network enables efficient phonon transport via low-resistance pathways. (2) Thermal permeability theory predicts a λ jump at φ_c_, but this occurs only in specific systems (e.g., high-aspect-ratio fillers), with universality constrained by filler morphology and dispersion. (3) Thermal elasticity coefficient theory, leveraging dynamical similarity between phonon propagation and mechanical vibration, analogizes λ to thermal vibration’s equivalent elasticity coefficient; high-λ fillers enhance performance via a “thermal enhancement effect.” Nonetheless, phonon scattering at heterogeneous interfaces remains a core bottleneck, requiring synergistic suppression of interface mismatch via surface modification and topological optimization.

Filler configuration regulation, benefiting from suppressed interface scattering, improved defect resistance, and network construction, yields anomalous λ patterns, with multi-layer GO outperforming single-layer. Optimization requires synergizing large low-defect fillers, oriented network construction, and surface modification to reduce intrinsic scattering and interface resistance, overcoming threshold limitations [15,19,106].

#### 4.1.5. Interfacial Thermal Resistance of Composite Materials

The interface transition zone of polymer composites serves as a thermal barrier due to chemical composition and microstructural gradients. Core barrier Kapitza thermal resistance (R_k_) arises from phonon vibration mismatch, microstructural defects, and weak interface coupling, constraining phonon transmission efficiency [16,19,34].

Interfacial thermal resistance suppresses heat conduction via two pathways. At matrix-filler interfaces, phonon transmission efficiency η is quantified by the acoustic mismatch model (AMM): η = 4Z_1_Z_2_/(Z_1_ + Z_2_)^2^. At filler-filler interfaces, contact thermal conductivity Gc follows a power law with contact pressure. This dual resistance causes effective thermal conductivity k_eff_ to deviate from the ideal mixing rule k_eff_/k_m_ = 1 + β_φ_/(1 − γ_φ_), where shape factor β and interfacial resistance coefficient γ determine system behavior [15,107].

The size effect is quantified by the Kapitza radius (a_k_) [15,108]: a_k_ = R_k_·k_m_. When filler size a > a_k_, k_eff_ increases; otherwise, interfacial resistance dominates (k_eff_ < k_m_), explaining multi-layer graphene’s advantage over single-layer at low loads.

Current research identifies three theoretical frameworks. First, lattice dynamics-based phonon transport models (AMM, DMM). AMM treats phonons as elastic plane waves with specular reflection/refraction at smooth interfaces (neglecting inelastic scattering). DMM assumes full diffusion scattering, with phonon transmittance proportional to DOS_f_/DOS_m_ (detailed balance) [97,109]. Both models fail to quantify interface microstructure. Second, equivalent medium theory (EMT) models: Hashin-Shtrikman (H-S), Hamilton-Hasselman (H-H), Foygel. H-S provides variational bounds for interfacial thermal resistance ($R_{d}^{*}$), handling multiphase fillings but ignoring local contact (Equation (4)) [15,110,111].

(4) $$\frac{\lambda_{c}}{\lambda_{m}}=\frac{1+2\left({13.3347e}^{-13.2701R_{d}^{*}}\right)K\sum_{i=1}^{n}E_{i}V_{i}}{1-\left({13.3347e}^{-13.2701R_{d}^{*}}\right)\sum_{i=1}^{n}E_{i}V_{i}}$$

The H-H model enhances Maxwell’s theory by incorporating shape factor *n* to compute interfacial thermal resistance for irregular fillers (Equation (5)) [15,112,113].

(5) $$\frac{\lambda_{c}}{\lambda_{m}}=\frac{\lambda_{f}\left[1+\left(n-1\right)\frac{R_{d}\lambda_{m}}{\alpha }\right]+\left(n-1\right)\lambda_{m}+\left(n-1\right)V_{f}\left[\lambda_{2}\left(1-\frac{R_{d}\lambda_{m}}{\alpha }\right)-\lambda_{m}\right]}{\lambda_{f}\left[1+\left(n-1\right)\frac{R_{d}\lambda_{m}}{\alpha }\right]+\left(n-1\right)\lambda_{m}+V_{2}\left[\lambda_{f}\left(1-\frac{R_{d}\lambda_{m}}{\alpha }\right)-\lambda_{m}\right]}$$

In comparison, the Foygel model focuses on the thermal resistance of the packing network, using predictions corrected by the overflow threshold and network connectivity. The third category, cross-scale simulation methods, employs MD (coupled with the Green-Kubo formula) to quantify interfacial phonon scattering and FEA to solve the heat diffusion equation for modeling microstructures with pores and grain boundaries [114,115]. Table 3 compares these models.

### 4.2. Preparation Process of High-Thermal-Conductivity Polymer Composite Materials

**Figure 6:**
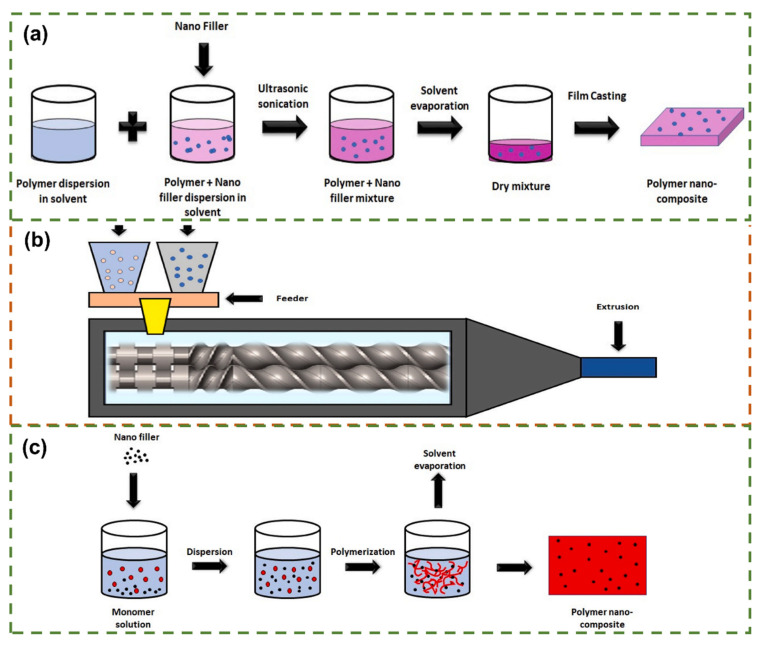
Schematic diagram of the three processes: (**a**) Solution blending method, (**b**) Melt blending method, and (**c**) In-situ polymerization method [22]. Copyright 2022, Elsevier.

The performance of high-thermal-conductivity polymer composites depends on filler network topology, as preparation processes directly determine thermal conduction path continuity and interface thermal resistance suppression. This section briefly overviews solution mixing, melt mixing, and in situ polymerization methods, analyzing their characteristics and advantages. Figure 6 illustrates these three process flows.

#### 4.2.1. Solution Mixing Method

Solution mixing, a liquid–phase dispersion strategy, is critical for nanoscale graphene dispersion in polymer matrices. Its core process involves three steps: precursor functionalization, co-solvent compounding, and in situ molding [19,34]. First, modified GO is dispersed in a polar solvent; chemical or thermal reduction yields single-layer-dominated RGO suspensions. Second, RGO and polymers are sheared in a common solvent, with π-π and van der Waals interactions driving uniform dispersion. Finally, 3D composites form via casting, spin-coating, or solvent evaporation [34]. Advantages include high dispersion efficiency, controlled component distribution, and short cycles, positioning it as a promising technique.

**Figure 7:**
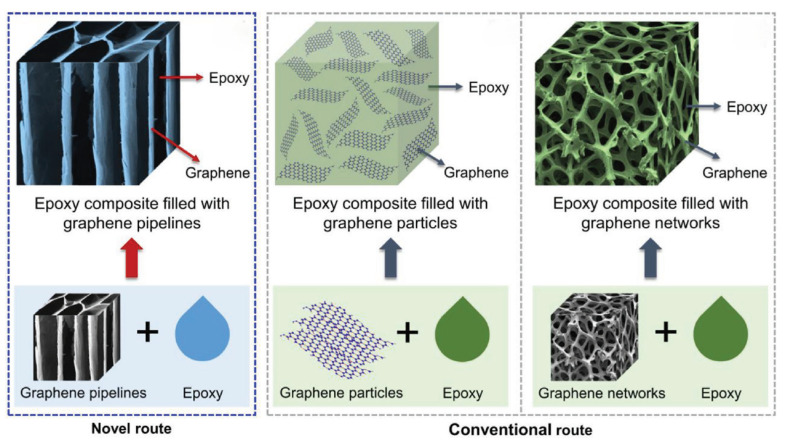
Design scheme for constructing an efficient heat-transfer channel in graphene/epoxy composite materials [14]. Copyright 2024, John Wiley and Sons.

Ma et al. [14] combined solution mixing with chemical/thermal reduction to prepare GO/EP composites (Figure 7). By reconstructing graphene’s sp^2^ conjugation and forming efficient thermal networks, the composite achieved 69.74 W·m^−1^·K^−1^ at 11.22 wt.% filler, overcoming traditional filler loading limits and offering new pathways for high-performance electronic thermal management materials. Fan et al. [116] used an improved Hummers method to control GO oxygen-functional group density, enabling oxidation degree optimization. This strategy enhanced room- and low-temperature tensile properties, with a 31% strength increase. Moderate oxidation improved interfacial interactions, but excessive oxidation weakened graphene structure and reduced performance, revealing oxidation degree-structure-performance correlations to guide high-performance nanocomposite design. Sharif et al. [117] prepared chitosan-graphene oxide (CGO) hybrids and epoxy nanocomposites via solution mixing. CGO’s 3D structure and enhanced matrix interfacial interactions improved epoxy composites’ mechanical and thermal properties: 65% tensile modulus, 56% tensile strength, and 21 °C glass transition temperature increase. This clarified CGO’s synergistic enhancement mechanism, providing a new interface engineering strategy for high-performance polymer nanocomposites.

#### 4.2.2. Melt Blending Method

The melt blending method, a key, widely adopted technique for preparing graphene-based high-thermal-conductivity polymer composites, is ideal for large-scale production. As a solvent-free solid-state dispersion technique, it achieves nanoscale GO dispersion in thermoplastic matrices via thermomechanical energy. At the polymer’s viscous flow temperature, the twin-screw extruder’s high shear field overcomes interlayer van der Waals forces, exfoliating and dispersing graphene [52,97]. Advantages include short process flow, controllable energy, environmental friendliness, and strong industrial adaptability. However, high temperatures may induce sp^2^ structural defects, flake fragmentation, and increased system viscosity, reducing dispersion uniformity and limiting thermal conductivity enhancement [22]. Inherent poor graphene-polymer interfacial compatibility increases thermal resistance, a critical limitation [97]. To address these, researchers use GO surface modification, multi-component synergism, and process parameter optimization to enhance thermal conductivity and interfacial interactions.

#### 4.2.3. In Situ Polymerization Method

In situ polymerization is a key technique for preparing graphene-based high-thermal-conductivity polymer composites. It involves dispersing graphene derivatives in polymer monomers/prepolymers to form a premixed system, then using an initiator to catalyze in situ polymerization at the filler interface, embedding GO in the matrix during polymerization to yield nanocomposites with stronger interfacial bonding [15,52]. Compared to melt blending, it offers dual advantages [118,119]: covalent/non-covalent grafting between polymer chains and graphene layers enhances interfacial interactions via grafted structure compatibility. It also regulates filler dispersion and interfacial bonding, mitigating agglomeration from surface energy differences in traditional processes, providing a molecular-level strategy to enhance composite thermal conductivity.

**Figure 8:**
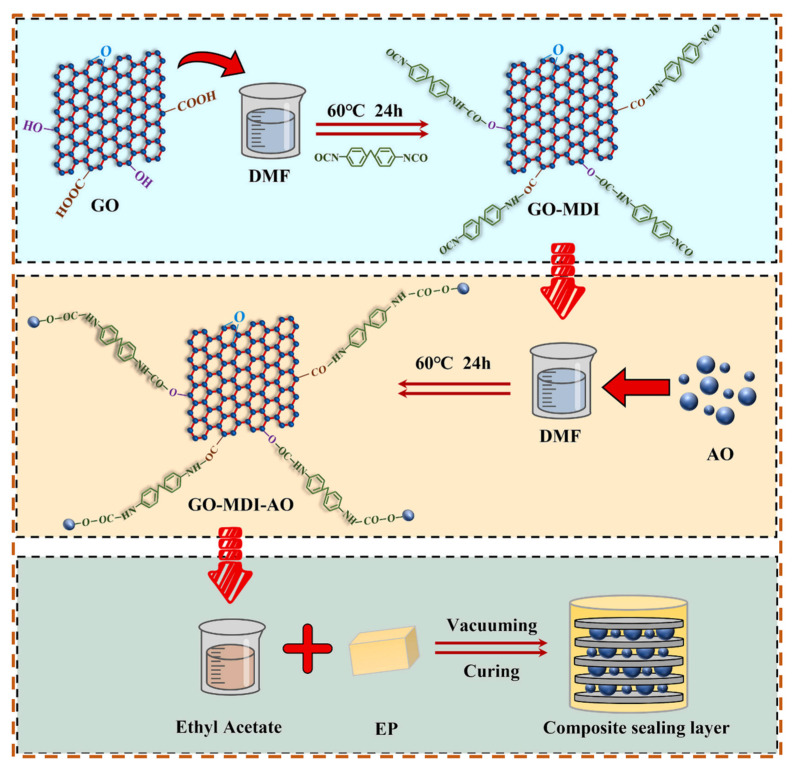
Schematic diagram of the preparation of GO-MDI-AO/EP composite material [121]. Copyright 2024, Elsevier.

Luo et al. [120] prepared melamine resin/graphene hybrids via in situ polymerization, then combined them with EP using vacuum-assisted molding to produce anisotropic, thermally conductive, flame-retardant composites. The material showed anisotropic thermal conductivity (1.56 W·m^−1^·K^−1^ horizontal, 0.40 W·m^−1^·K^−1^ vertical), a 51% lower peak heat release rate, and synergistically improved flame retardancy. Qiu et al. [121] used 4,4′-diphenylmethane diisocyanate as a coupling agent to in situ construct a 3D binary filler network of GO and Al_2_O_3_, then gradient-cured it to form a thermally conductive EP composite (Figure 8). With 10% binary filler, the epoxy sealant’s thermal conductivity increased by 118.75%, gas barrier by 24.63% and ultimate displacement by 32.77%, due to a 2D-spherical structure synergistically forming thermal/barrier networks and inducing phase transformation toughening.

However, in situ polymerization has limitations: interface modification may obscure graphene’s conjugated structure, hindering electron transport. Sharp viscosity increases degrade rheological properties, requiring precise polymerization kinetics control to balance dispersibility and processability. Modifier side reactions also pose risks, necessitating real-time monitoring for performance control [22,122].

### 4.3. Effect of Different Preparation Methods on Composite Material Properties

GO/EP composites exhibit significant potential in electronic packaging and thermal management owing to their superior thermal conductivity. Preparation methods critically influence GO dispersion in the EP matrix, interfacial interactions, and final thermal conductivity. Below, we analyze the effects of the three aforementioned methods on preparing high-thermal-conductivity GO/EP composites, focusing on three aspects: GO dispersion, interfacial interactions, and thermal conductivity.

#### 4.3.1. Graphene Dispersibility

Uniform GO dispersion in polymer matrices is critical for high-performance composites, as agglomeration—GO’s primary application challenge—severely degrades composite performance.

Solution mixing disperses GO in a solvent, mixes with EP, and controls evaporation/mixing to achieve good GO dispersion, enhancing composite thermal conductivity and mechanical properties [34,118]. Ultrasonication or dispersants mitigate GO agglomeration by overcoming van der Waals forces [123]. Yet, evaporation may induce re-agglomeration (notably at high GO loadings) [124], and residual solvents can compromise final performance.

Melt blending mixes GO and EP in a molten state via shear, avoiding solvents and aligning with industrial production needs. However, high polymer viscosity and strong interlayer graphene interactions hinder exfoliation and dispersion in the molten state. Poor graphene-polymer compatibility further induces micron- to macroscopic agglomerates [22,34], reducing GO utilization and limiting thermal conductivity enhancement. To address this, surface modification or process parameter adjustment enhances shear efficiency [125]. Yin et al. [126] used phase transition-assisted melt blending to exfoliate expanded graphite in situ, improving dispersion in low-density polyethylene and enhancing thermal conductivity.

In situ polymerization uses GO as a reaction/initiation site or template, achieving nanoscale uniform dispersion and enhancing composite performance [118,127]. In situ thermal reduction yields functionalized RGO with uniform dispersion in epoxy nanocomposites, significantly boosting performance [128].

#### 4.3.2. Interface Interactions

Strong interfacial interactions are critical for efficient heat transfer from the polymer matrix to graphene fillers, maximizing composite thermal conductivity.

In solution-mixed composites, interfacial interactions primarily arise from physical adsorption and weak chemical bonding between GO and EP [15,22]. GO’s inherent chemical inertness limits solvent compatibility and dispersibility [13], complicating processing and composite preparation. Grafting amine-rich compounds onto GO enhances compatibility with epoxy groups, optimizing interfacial interactions and enabling high-performance composites [129]. Condensation reactions grafting methylphenyl silicone intermediates onto EP further improve compatibility, stabilizing uniform GO dispersion and enhancing thermal, mechanical, and adhesive properties [130].

**Figure 9:**
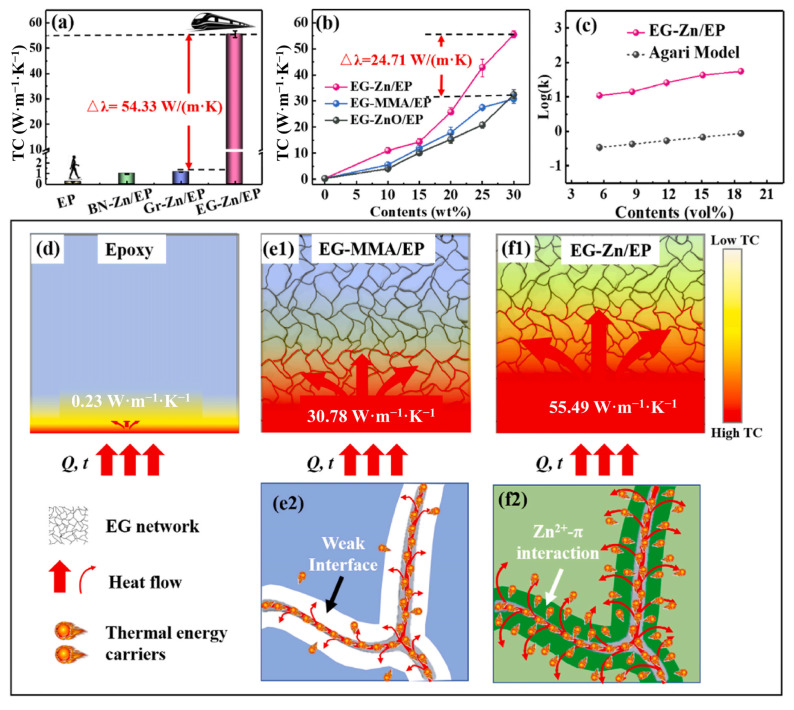
Thermal Conductivity and Mechanisms of EG-Zn/EP Composites. TC values for different fillers (**a**) and filler loadings (**b**), Agari model fitting curves (**c**), (**d**–**f2**) Thermal conduction mechanism models for epoxy resin and different composites [133]. Copyright 2023, Elsevier.

Melt blending relies on weaker forces (e.g., physical entanglement, van der Waals interactions) for GO-EP interfacial bonding. High mixing temperatures may break/rearrange GO surface bonds, reducing interfacial strength. Insufficient shear stress can create voids between GO and the matrix, increasing interfacial thermal resistance and limiting performance [131,132]. However, Zn^2+^ enhances π-bond interactions at expanded graphite-epoxy interfaces, boosting thermal conductivity to 55.49 W·m^−1^·K^−1^ [133], as shown in Figure 9.

In situ polymerization enables stronger interfacial interactions—even covalent bonding—by allowing polymer segments to grow on GO surfaces [134]. EP monomers polymerize on GO, forming tight chemical bonds or physical entanglements that enhance heat and force transfer efficiency, improving thermal conductivity and mechanical properties. Additionally, Diels-Alder-triggered self-healing EP nanocomposites, functionalized with graphene nanosheets, enhance flexibility and self-healing efficiency [135].

#### 4.3.3. Thermal Conductivity

Composite thermal conductivity reflects graphene dispersion and interfacial interactions. Despite GO’s high intrinsic λ, transferring its properties to macroscopic composites remains challenging.

In solution-mixed GO-polymer composites, λ increases linearly with filler content at low loadings. Beyond the threshold, van der Waals-induced agglomeration and increased interfacial thermal resistance cause nonlinear decay in enhancement efficiency, potentially plateauing or degrading performance due to filler-matrix delamination [136].

**Figure 10:**
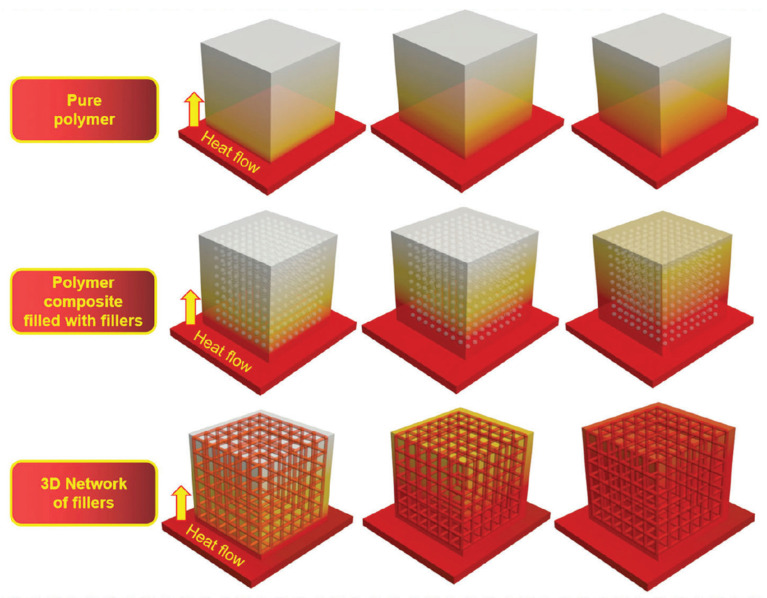
Schematic of a 3D heat conduction network for efficient heat transfer [97]. Copyright 2023, John Wiley and Sons.

Melt blending struggles to form thermal networks due to poor GO dispersion, yielding less significant λ improvements than solution mixing or in situ polymerization. Filler dispersion and connectivity are critical; even high graphene loadings only marginally enhance λ without effective conduction paths. Obviously, constructing a three-dimensional thermal conduction network can significantly improve thermal conductivity [69,97], as shown in Figure 10.

In situ polymerization achieves higher λ via uniform GO dispersion and strong interfacial interactions, forming continuous thermal networks for efficient heat transfer between GO layers and the matrix. Phosphorus-functionalized boron-nitrogen/GO hybrids yield λ = 0.95 W·m^−1^·K^−1^ at 4.04 vol% filler, a 377% increase over pure EP [137]. Table 4 details GO effects on epoxy λ.

In summary, in situ polymerization excels in GO dispersion and interfacial interaction, effectively enhancing composite λ. Melt blending, though promising for industrial production, faces dispersion and interface challenges; solution mixing works well for lab-scale/small batches but suffers from solvent residues and environmental issues in large-scale use.

## 5. Applications of High-Thermal-Conductivity Graphene/Epoxy Composite Materials

GO, with outstanding thermal conductivity, excellent mechanical strength, and low density, shows significant application potential in diverse fields. This section focuses on high-thermal-conductivity GO/EP composites, systematically discussing progress in their applications to cutting-edge fields such as electronic packaging heat dissipation, high-performance energy storage, and electromagnetic interference shielding. It emphasizes their critical role in enhancing thermal management efficiency of related systems, aiming to support technological development with theoretical and practical guidance.

### 5.1. Electronic Packaging Field

As electronic devices evolve toward higher power, miniaturization, and integration, thermal management has grown increasingly critical. Heat accumulation during operation degrades component performance and lifespan, heightening requirements for high-performance electronic packaging materials [1,14,42]. However, traditional epoxy packaging materials are inherently constrained by their low thermal conductivity. Incorporating GO to construct 3D thermal networks significantly improves thermal conductivity [18,97]. Thermal interface materials (TIMs) establish efficient heat conduction pathways between heat sources (e.g., chips) and heat sinks, thereby reducing contact thermal resistance. Owing to the low thermal conductivity of conventional electronic packaging materials (e.g., ceramics, metals), they struggle to meet heat-dissipation requirements (Table 2). In contrast, GO/EP composites exhibit multiple advantages: high thermal conductivity, excellent processability, compatibility with electronic components, and a lightweight nature. Due to the in-plane thermal conductivity of its 2D sheet structure and the capability to readily construct 3D thermal networks, GO/EP composites have emerged as a key material for next-generation electronic packaging.

Compared with boron nitride (BN, highly anisotropic, with in-plane thermal conductivity ~400 W·m^−1^·K^−1^ but out-of-plane thermal conductivity only 2–3 W·m^−1^·K^−1^) and carbon nanotubes (CNTs, one-dimensional structures prone to entanglement with low network formation efficiency), graphene can simultaneously enhance both in-plane and out-of-plane thermal conductivity via “layer stacking.” TIMs are interposed between heat-generating components and heat sinks. Their core function is to fill microscopic irregularities on contact surfaces and displace air (a poor thermal conductor), thereby reducing thermal contact resistance (TCR) and establishing efficient heat conduction pathways. Table 5 demonstrates that GO/EP composites exhibit not only excellent processability but also high thermal conductivity.

**Figure 11:**
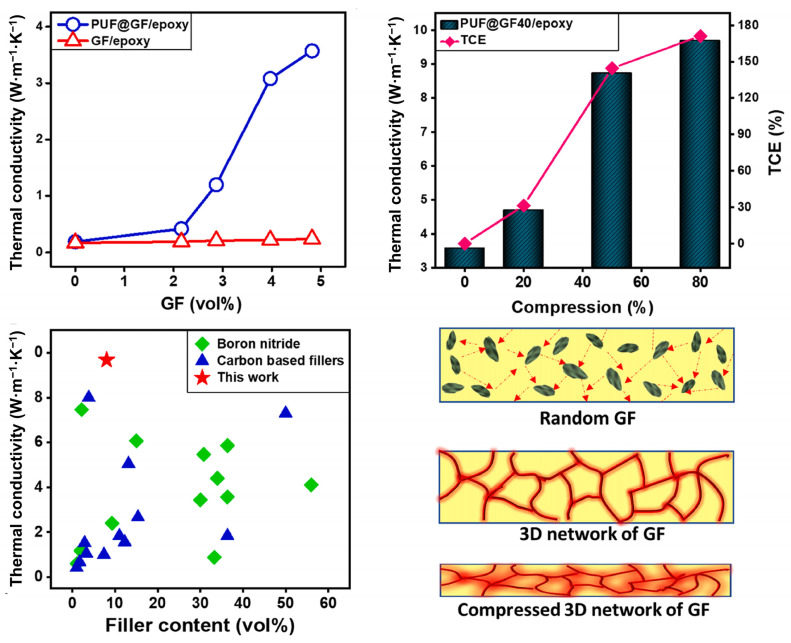
Thermal conductivity, compressive strength enhancement, thermal conductivity comparison, and 3D-network heat-transfer mechanism of PUF@GF/epoxy composites [142]. Copyright 2021, Elsevier.

To enhance EP’s corrosion resistance and thermal performance, researchers [147] added BaSO_4_ powder and RGO, achieving thermal diffusivity of 51.46 mm^2^·s^−1^ (2 wt% RGO, λ = 133.0 W·m^−1^·K^−1^) and 71.38 mm^2^·s^−1^ (5 wt% filler, λ = 160.0 W·m^−1^·K^−1^). Aluminum nitride (AlN), with high thermal conductivity and insulation, was combined with polythiophene/graphene complexes via ultrasonic exfoliation to reinforce epoxy films. Only a small complex addition significantly improved EP’s λ [148]. Fluorinated graphene (FG), with unique C–F covalent/ionic bonds, outperforms other derivatives [35]. Mani et al. [142] prepared FG via ball milling, then used electrostatic assembly and vacuum impregnation to create epoxy composites with λ = 9.68 W·m^−1^·K^−1^, demonstrating thermal management potential (Figure 11). EP’s 3D cross-linked structure makes traditional recycling difficult. Kang et al. [143] addressed this by using waste EP powder to induce 3D graphite nanosheet networks, producing ultrahigh-thermal-conductivity composites (λ = 10.1 W·m^−1^·K^−1^) with enhanced thermal management and electromagnetic shielding, offering a strategy for thermoset upcycling.

Traditional single-filler systems are susceptible to a “performance ceiling” effect due to their inherent physicochemical properties, characterized by mutually restrictive mechanical strength, thermal conductivity, and processability parameters, along with performance degradation beyond the percolation threshold. Therefore, the four core mechanisms of multi-scale hybrid filler design—percolation synergy, defect compensation, interface phonon matching, and functional integration optimization—were systematically summarized. These mechanisms’ capability to enable multifunctionalization of composite materials through the construction of synergistic enhancement networks was analyzed, and innovative solutions were proposed for applications including electronic packaging and thermal management. The limitations of single-filler systems are manifested in graphene’s tendency to agglomerate, boron nitride’s limited in-plane orientation, and ceramic fillers’ high interfacial thermal resistance. These issues result in a negative correlation among multiple key parameters (mechanical strength, thermal conductivity, and processing fluidity) of composite materials beyond the percolation threshold. Therefore, urgent development of novel design strategies is required to overcome this bottleneck.

By exploiting the spatial complementarity and functional synergy of heterogeneous fillers, the performance limitations of single-filler systems are addressed. Ju et al. [149] achieved a thermal conductivity of 0.87 W·m^−1^·K^−1^ in a 60 µm thick epoxy coating using a 30 wt% BN + 0.5 wt% GNP-PVP stereochemical barrier network. Notably, this system maintained an impedance of 6.5 × 10^9^ Ω·cm^2^ even after 7 days of immersion. Zhang et al. [150] constructed a graphene-coated BNNS three-dimensional framework using combustion-ice templating. At a filler loading of 11.2 vol%, the in-plane thermal conductivity reached 2.23 W·m^−1^·K^−1^ (room temperature) and 1.09 W·m^−1^·K^−1^ (77 K), respectively, representing a 1073% enhancement and enabling efficient heat dissipation for high-power LEDs and 3D integrated chips. Targeted modification of fillers’ inherent defects enables precise mitigation of performance shortcomings. Li et al. [151] encapsulated liquid metal with graphene oxide and stress-oriented the composite, achieving a 2.34-fold increase in KIC for 11.2 wt% LMGF/epoxy, an EMI SE of 48 dB, and a photothermal de-icing efficiency of 52%, thereby satisfying the multifunctional requirements of aviation applications. Enhancing phonon transport via molecular-level interface regulation improves thermal conduction efficiency. Sun et al. [152] utilized π-π coupling between PDA-G and CNF hydrogen-bonded interfaces, achieving a vertical thermal conductivity of 0.58 W·m^−1^·K^−1^ at 3.05 wt% filler loading and reducing the coefficient of thermal expansion (CTE). Wang et al. [153] fabricated vertically aligned graphene aerogel using a “fire-ice” non-freeze-drying process combined with a 2850 °C graphitization process. This achieved a vertical thermal conductivity of 11.6 W·m^−1^·K^−1^ at 1.84 vol% filler loading in epoxy (a 3640% efficiency enhancement), a 2.6-fold increase in compressive strength, and an EMI shielding effectiveness (EMI SE) of 40 dB, thereby realizing thermal-electromagnetic integration. Integrating external stimuli with filler characteristics enables high-performance output in specific application scenarios. Lv et al. [154] employed a CTAB-Al_2_O_3_@graphene insulating layer to achieve a thermal conductivity of 0.97 W·m^−1^·K^−1^ in 30 wt% epoxy composites, while maintaining a volume resistivity of ≥10^11^ Ω·cm. Yan and Wu [155,156] utilized the magnetic properties of GNPs under a bidirectional magnetic field or GR-Fe_3_O_4_ under planar orientation, achieving 276.6%/54.8% enhancements in internal thermal conductivity at 5/2 wt% loadings while maintaining insulation >10^14^ Ω·cm.

In summary, by integrating the intrinsic thermal conductivity of GO with the insulation and processability advantages of EP, a multi-scale hybrid design enables simultaneous addressing of mutually restrictive multi-objective parameters (thermal conductivity, insulation, mechanical properties, and processability), thereby offering a green, efficient, and scalable thermal management solution for next-generation electronic packaging. Future research should prioritize the development of dynamic-responsive intelligent filler networks and design for adaptability to extreme environments, driving the advancement of polymer composites toward higher performance and multifunctionality.

### 5.2. Energy Storage Field

In energy storage systems, thermal management efficiency directly influences battery cycle life and system safety. High-thermal-conductivity composite materials, via multi-scale heat flow path optimization and phase change heat-transfer augmentation, have emerged as core materials for overcoming traditional thermal management bottlenecks [19,97]. GO integrates two-dimensional high-thermal-conductivity channels with chemically tunable interfaces. On one hand, GO can be designed as a three-dimensional GO/EP framework within battery modules, constructing low-thermal-resistance pathways along the thickness direction and thereby reducing local hotspot temperature rise by 30–50%. On the other hand, when blended with paraffin- or fatty acid-based phase change materials (PCMs), GO forms a continuous thermal conduction network, enhancing effective thermal conductivity by an order of magnitude. Meanwhile, the layered barrier effect of GO significantly mitigates the leakage of the phase change medium. This offers a scalable material solution for integrated battery-energy storage systems that incorporate both high energy density and long lifespan.

#### 5.2.1. Challenges and Applications of Battery Thermal Management Systems

As a key material in battery thermal management, GO/EP composite materials enhance thermal management efficiency and battery pack safety via multi-scale interface engineering and functional design. Lithium-ion batteries, serving as the core power source for electric vehicles and hybrid vehicles, are well documented to be highly sensitive to temperature variations in performance and lifespan [157]. Lithium-ion batteries generate substantial waste heat during rapid charge-discharge cycles. Localized overheating not only accelerates battery aging and shortens cycle life but may also trigger severe safety incidents (e.g., thermal runaway). Elevated temperatures or temperature gradients can readily induce performance degradation, diminished lifespan, and thermal runaway hazards. Thus, the development of efficient battery thermal management systems is critical for preserving battery performance and prolonging lifespan. GO/EP composite materials can be employed as potting compounds or thermal pads or incorporated into liquid/air cooling systems to enable precise temperature regulation of battery packs.

Polymer-based composite bipolar plates (CBPs) enhance conductivity, thermal conductivity, corrosion resistance, and mechanical properties via 3D conductive networks, overcoming traditional material conductivity limitations and enabling multi-performance optimization for stable equipment operation [144,146]. A graphite/epoxy CBP with a 3D conductive network, prepared by regulating graphite content to optimize the network, achieves 212.64 S·cm^−1^ in-plane electrical conductivity and 16.01 W·m^−1^·K^−1^ thermal conductivity [144]. Exhibiting 317.52% higher power density than traditional composites, it also shows excellent corrosion resistance and hydrophobicity, offering a new strategy for high-performance polymer-based CBPs. Chen et al. [158] optimized phenolic resin networks via copolymerization with epoxy resin flexible segments (−CHOH−CH_2_−O−), achieving 46.2 MPa bending strength with high conductivity (188 S·cm^−1^), low interfacial resistance (3.32 mΩ·cm^2^), and corrosion resistance, advancing PEMFC practical application. A three-stage optimized copper fiber/carbon black/graphite-epoxy CBP, incorporating 8 vol% copper fibers into a 40 vol% epoxy matrix, achieves 169 S·cm^−1^ conductivity, 43 MPa bending strength, and 17 W·m^−1^·K^−1^ thermal conductivity, meeting DOE-2025 corrosion and power density criteria [145].

However, lithium-ion battery systems encounter significant challenges related to temperature sensitivity during practical operation, as their electrochemical behavior and thermal properties exhibit strong temperature dependence: Electrode reaction kinetic parameters (e.g., lithium-ion diffusion coefficients and charge transfer rates) increase exponentially with temperature elevation, yet this also exacerbates the likelihood of side reactions. Battery internal resistance exhibits nonlinear fluctuations owing to variations in electrolyte viscosity and the temperature sensitivity of electrode/electrolyte interface impedance. The stability of the solid electrolyte interphase (SEI) is particularly temperature-sensitive. In elevated-temperature environments, the balance between dynamic repair and rupture of the SEI is disrupted, accelerating irreversible capacity degradation. More critically, battery systems carry an inherent risk of thermal runaway. Under rapid charge-discharge conditions, local hot spot formation induces heat accumulation, triggering a chain of exothermic reactions: at elevated temperatures, the cathode material undergoes oxidation and decomposition, releasing copious oxygen that reacts violently with the flammable organic electrolyte. This process further exacerbates temperature increases, forming a self-perpetuating vicious cycle that may ultimately result in catastrophic failures (e.g., battery swelling, fire, or explosion).

#### 5.2.2. Multi-Scale Mechanisms and Synergistic Optimization of Phase Change Energy Storage Materials

PCMs are a class of functional materials utilizing latent heat storage mechanisms. However, their practical application has long been constrained by inherent thermophysical limitations. During solid–liquid phase change, liquid–phase fluidity readily induces encapsulation failure and PCM leakage. Moreover, inherently low thermal conductivity significantly limits the heat-transfer rate during energy storage/release, leading to energy conversion efficiency far below theoretical predictions. To address this bottleneck, researchers have focused on developing solid–liquid phase change systems with high enthalpy, leveraging their capacity to absorb or release substantial latent heat during phase change for efficient self-regulating thermal management. Concurrently, through designing composite reinforcement structures and adopting precision encapsulation techniques, they have effectively addressed volume expansion and leakage during phase change, significantly enhancing the material’s morphological stability and structural integrity. This design strategy, integrating high-enthalpy phase change properties with advanced encapsulation structures, not only enhances the thermophysical performance of PCMs but also offers robust technical support for their application in dynamic thermal management, intelligent temperature control, and related applications.

**Figure 12:**
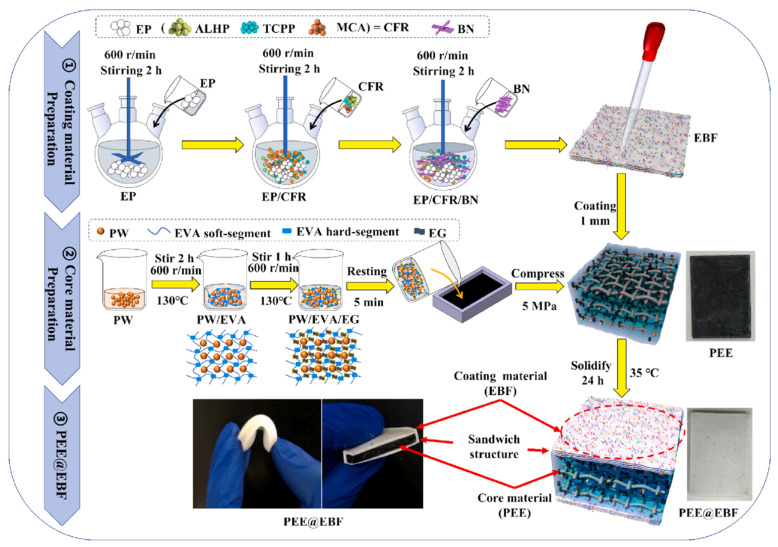
Preparation process of sandwich-type phase change material [159]. Copyright 2025, Elsevier.

Shen et al. [159] developed a sandwich-type flame-retardant flexible PCM (PEE@EBF) with 166.6 J·g^−1^ latent heat, 0.8 W·m^−1^·K^−1^ thermal conductivity, and UL94V-0 flame retardancy, reducing battery module peak temperature by 11.8 °C and delaying thermal runaway by 633 s via a chemical-physical synergistic flame-retardant mechanism. Wood-based composites, prepared via delignification, graphene modification, and PCM impregnation, achieve 218.5 J·g^−1^ phase change enthalpy and synergistic electrothermal conversion (Figure 12). Wood pores drive Joule heat for dynamic energy storage, offering a sustainable thermal management solution for electronics [160]. A 3D composite PCM, using a gradient porous graphite foam skeleton loaded with stearic acid, synergistically enhances thermal conductivity and compressive strength, achieving 99.9% energy storage efficiency and 200-cycle thermal stability [161]. Huang et al. [162] fabricated a leak-proof, vibration-resistant flexible composite PCM by reinforcing a cross-linked ethylene-butadiene-styrene block copolymer framework with ternary ethylene-propylene rubber. It shows >99% mass retention after 60 °C/10 h testing, <50 °C temperature rise, and <2 °C temperature difference during 1C battery module discharge, enabling dual leak prevention and vibration resistance for passive thermal management.

PCMs in GO/EP composite systems exhibit notable advantages in thermal conductivity enhancement and leak prevention; however, their practical application still confronts multifaceted technical bottlenecks and scientific challenges [163,164,165]. First, thermal conductivity enhancement is limited by GO layer aggregation, which requires surface modification or solvent blending to improve dispersion. However, achieving uniform dispersion while controlling costs remains challenging under high filler loadings and excessive filler addition can induce brittleness. Secondly, leakage induced by solid–liquid phase transitions is dependent on encapsulation design yet requires balancing fluidity and long-term shape stability. Microencapsulation technology or dynamic covalent bonds (e.g., disulfide bonds) can be utilized to improve structural integrity. Thirdly, GO incorporation can reduce phase change enthalpy, which requires adjusting component ratios and interfacial interactions to synergistically enhance thermal energy storage performance. Fourthly, cyclic stability is affected by epoxy aging, interfacial delamination, and thermal fatigue, which demands cross-scale design to synergistically enhance thermal conductivity, thermal energy storage, and durability.

### 5.3. Electromagnetic Shielding Field

With the rapid advancement of 5G communications, the Internet of Things (IoT), and future smart technologies, electromagnetic radiation pollution has become an increasingly significant global challenge. It not only poses potential threats to human health but may also disrupt ecosystem balance and severely impact the operational reliability of critical electronic equipment [166]. Long-term exposure to electromagnetic radiation may induce multi-system dysfunction and have adverse effects on living organisms. In the domain of electronic devices, electromagnetic compatibility (EMC) has increasingly emerged as a core design criterion, particularly amid the trends of high frequency and high integration. Electromagnetic interference (EMI) has become a key factor limiting electronic devices’ performance and stability, and could even result in device failure. Therefore, the development of high-performance electromagnetic shielding materials (ESMs) has become an urgent requirement to mitigate this challenge.

The core function of electromagnetic shielding materials lies in their ability to effectively attenuate or block electromagnetic wave propagation via diverse mechanisms, thereby safeguarding sensitive equipment and lowering environmental radiation levels. These mechanisms primarily encompass reflection loss (SE_R_), absorption loss (SE_A_), and internal multiple reflection loss (SE_M_). During the development of novel high-performance electromagnetic shielding materials, GO-based composites exhibit significant potential, particularly those with high thermal conductivity (e.g., GO/EP composites). GO is distinguished by its exceptional electrical conductivity, thermal conductivity, mechanical strength, and ultrahigh specific surface area, rendering it an ideal constituent for fabricating high-efficiency electromagnetic shielding materials. Through the incorporation of GO into an epoxy resin matrix, a conductive network is formed, enhancing dielectric loss capabilities and enabling multiple reflection effects at internal interfaces, thus effectively suppressing electromagnetic wave propagation. Furthermore, GO’s intrinsic high thermal conductivity empowers these composites to tackle both EMI concerns in electronic devices and thermal management needs—a feature particularly critical for high-power, miniaturized electronic devices.

**Figure 13:**
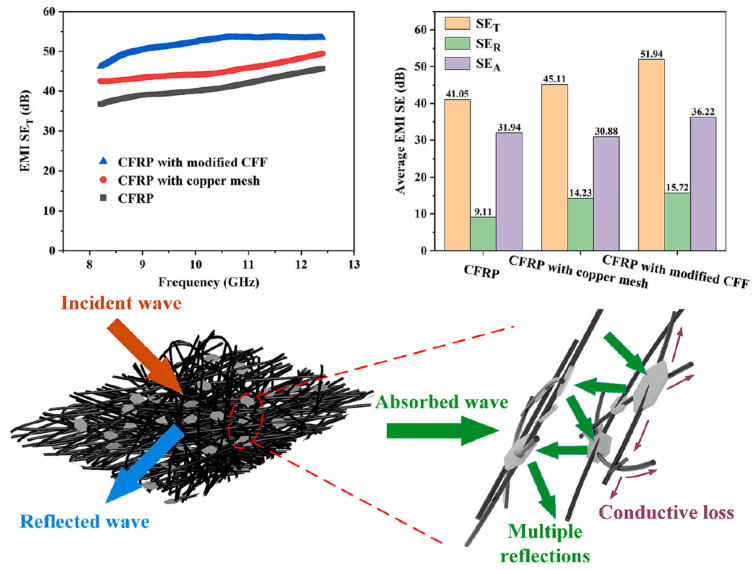
EMI SE_T_, average EMI SE, and shielding enhancement mechanism of composites [138]. Copyright 2023, Elsevier.

Currently, electromagnetic shielding materials are being developed in the direction of being lightweight, flexible, thin, multifunctional, and environmentally friendly. Polyacrylonitrile-based carbon fiber felt (CFF), widely used for high-temperature insulation post-annealing, has discontinuous conductive networks due to its chopped structure, limiting EMI application potential. Zhu et al. [138] prepared composites via Joule-heating-induced GO-CF co-crystallization and lamination, increasing thermal conductivity by 1.8 times and achieving 51.94 dB X-band EMI shielding effectiveness, surpassing commercial copper mesh and showing significant application potential (Figure 13). Jia et al. [139] synthesized NiCo-GNS hybrids via in situ growth, constructing 3D EP/PVDF/NiCo-GNS honeycomb skeletons via vacuum impregnation. With 15 wt% filler, epoxy composites showed 3.83 times higher λ and 34.62 dB X-band shielding, enabling synergistic thermal management and EMI shielding. Notably, 3D printing constructs functionalized polymer-based lightweight porous structures, with multi-scale regulation enabling efficient multifunctional-mechanical configurations for electronics. Du et al. [167] combined SLA-3D printing and vacuum impregnation to prepare polydimethylsiloxane (SGP)/epoxy acrylic resin composites integrating thermal conductivity, EMI shielding, and mechanical properties. The resin skeleton bears the load, while SGP provides thermal conductivity (2.13 W·m^−1^·K^−1^ at 12.4 GHz) and 45.93 dB EMI shielding. Applying an external magnetic field induces nanoparticle response, enabling oriented arrangement to attenuate electromagnetic waves via hysteresis loss, eddy current loss, and natural resonance. Jia et al. [168] prepared magnetically responsive cobalt@graphene nanosheets (Co@GNP)-reinforced epoxy via coprecipitation, constructing a magnetic field-oriented network for high-temperature thermal management. Validating heat-transfer models via four-parameter fitting and enthalpy change methods, synergistic optimization achieved 14.90 dB EMI shielding, a 419% improvement over pure EP.

As illustrated in Table 6, distinct nanomaterials demonstrate unique advantages in the domains of electromagnetic shielding and thermal conductivity enhancement. GO, characterized by low density, ultrahigh thermal conductivity, and a two-dimensional layered structure, exhibits 20–70 dB absorption-type shielding performance in high-frequency ranges (X-band and above). Its large specific surface area facilitates the formation of multiple scattering interfaces, rendering it suitable for flexible devices and transparent shielding applications. Carbon nanotubes exhibit 40–50 dB broadband absorption (1–18 GHz) via a three-dimensional conductive network; however, agglomeration issues require mitigation to optimize their application in aerospace. Metal fillers primarily depend on a 40–60 dB reflection mechanism in low-frequency ranges (below K-band); despite their high conductivity, their applicability in lightweight applications is constrained by high density and corrosiveness. Boron nitride, an insulator with shielding efficiency <20 dB, is exclusively utilized for thermal enhancement and necessitates conductive fillers to satisfy the insulation demands of 5G devices and LED packaging. In summary, performance discrepancies among these systems originate from their intrinsic conductivity, microstructure, and interfacial interactions, thereby offering multidimensional material choices for electronic packaging and thermal management.

### 5.4. Other Areas

Beyond prior applications, high-thermal-conductivity GO/EP composites exhibit potential in flame retardancy, anti-corrosion coatings, and aerospace, enhancing flame retardancy and corrosion resistance and enabling lightweight, high-thermal-conductivity aerospace components.

For flame retardancy, GO and flame retardants synergistically improve EP’s flame retardancy and thermal conductivity. A dense surface carbonized layer isolates oxygen/heat, delaying flame spread [172]. Chen et al. [173] prepared FeHP@GO hybrids via in situ self-assembly, using GO’s barrier effect, FeHP-catalyzed carbonization, and PO·/HPO· radical quenching to achieve triple optimization: LOI increased 42.5% (UL-94 V-0), PHRR decreased 46.2%, tensile strength rose 32.6%, and thermal conductivity improved 96.0%. Adding 3 wt% 1-butyl-3-methylimidazole tetrafluoroborate-functionalized Mo-MOF/GO hybrids enhanced flame retardancy (LOI = 27.6%, UL-94 V1; CO, PHRR, and smoke rates reduced by 55.88%, 52.10%, and 40.12%) with unchanged mechanical properties, via carbon-gas phase dilution-barrier effects [174].

For anti-corrosion coatings, GO provides physical barriers, chemical passivation, and thermal management, enhancing corrosion resistance. Yang et al. [140] blended RGO with BTA@HMS in epoxy, achieving 1.239 W·m^−1^·K^−1^ thermal conductivity (596% vs. pure epoxy) and a 4-order corrosion resistance improvement with 5 wt% filler. Yung et al. [147] prepared RGO/BaSO_4_ hybrid epoxy; 5 wt% BaSO_4_ yielded 165.0 W·m^−1^·K^−1^ in-plane thermal conductivity and 71.38 mm^2^·s^−1^ diffusivity for aluminum alloy protection. Liu et al. [175] optimized modified GO/PVB/epoxy coatings via the response surface method, achieving a 0.218 °C·s^−1^ heating rate and an 85.75° contact angle, confirming thermal/hydrophobic synergism.

In aerospace, GO/EP composites address lightweight, extreme-condition demands with high thermal conductivity, specific stiffness, and radiation resistance, enhancing thermal management and enabling high-power electronic packaging [21]. A GrF/Cu-CF/GrF sandwich composite, prepared by vertically weaving copper wires into carbon fiber fabric and laminating graphite fibers via vacuum-assisted resin transfer molding, achieved λ = 1.097 W·m^−1^·K^−1^ (104% vs. CFRP), attributed to copper/graphite conductivity and continuous heat pathways [115]. Graphene nanoplatelets/h-BN-modified epoxy nanocomposites, prepared via three-roll milling, synergistically enhanced conductivity, flexural strength, and thermal conductivity, meeting aerospace electrostatic dissipation and thermal management needs [176].

Additionally, high-thermal-conductivity GO/EP composites encounter performance degradation challenges attributed to the synergistic effects of hygrothermal aging and thermal cycling. Water penetration induces hydrolysis of ester bonds within the matrix, resulting in the breakdown of the cross-linked network and the weakening of mechanical properties. GO interface de-anchoring induces local failure of the three-dimensional thermal conduction network, while interfacial phonon scattering exacerbates thermal resistance increase. Furthermore, the thermal expansion coefficient mismatch between GO and EP induces shear stress during thermal cycling, further degrading interfacial bonding. In response, researchers have proposed multidimensional strategies: Du et al. [177] constructed a self-healing UV-shielding layer via in situ growth of FGO@CeO_2_ nanoparticles, significantly prolonging outdoor service life. Li et al. [178] revealed via molecular dynamics simulations that the GO/EP interface is more susceptible to water erosion, providing a foundation for atomic-level interfacial design. Xie et al. [179] demonstrated that multi-vacancy graphene strengthens interfacial bonding and optimizes thermal oxidation resistance. Mishra et al. [180] confirmed that hygrothermal environments can counteract the initial enhancement effect of graphene, emphasizing the significance of environmental durability design. These studies offer theoretical guidance and technical pathways for the development of long-lasting GO/EP composites.

## 6. Conclusions and Outlook

High-thermal-conductivity GO/EP composites are pivotal for addressing thermal management in electronics, with research establishing a framework for synergistic structure-performance-mechanism optimization. This review systematically summarizes their progress, elucidating core principles of thermal conductivity enhancement (percolation networks, interfacial phonon coupling), structural regulation strategies, and application challenges. GO’s thermal conductivity mechanisms depend on filler morphology, dispersion, and processing. Physical dispersion improves graphene distribution; chemical modification and in situ polymerization enhance interfacial bonding via surface functionalization, reducing thermal resistance. These composites advance electronic packaging, energy storage, and electromagnetic shielding, providing theoretical foundations and expanding practical applications.

Despite progress, challenges remain, requiring clarified future directions:

(1) Cross-scale structure design and dynamic characterization: Advanced characterization/simulation tools are needed to explore microscale thermal conductivity factors, build accurate models, and guide material design.
(2) Green/efficient preparation innovation: Key areas include innovative dispersion/chemical modification for optimal GO dispersion and interfacial interactions. Additive manufacturing (e.g., 4D printing) dynamically regulates thermal networks for flexible devices; machine learning optimizes processes to reduce energy/cost.
(3) Multi-physics coupling performance: Integrating thermodynamics, soft matter physics, and computational materials science enables a “composition-process-structure-performance” multi-scale prediction platform. Developing multifunctional composites (high thermal conductivity, impact resistance, and self-sensing) for 5G, AI hardware, flexible electronics, and extreme environments expands applications.

Future research should focus on performance improvement, cost control, and application expansion via interdisciplinary collaboration. Shifting from trial-and-error to theory-driven approaches, multidisciplinary innovation, and engineering breakthroughs will accelerate the lab-to-industry transition.

## Figures and Tables

**Table 2:** Thermal conductivity of polymers and fillers (W·m^−1^·K^−1^) [15,19,97]. Copyright 2025, Elsevier. Copyright 2025, Royal Society of Chemistry. Copyright 2023, John Wiley and Sons.

Polymer	λ	Filler	λ
EP	0.20	Au	345
PVDF	0.19	Ag	450
PMMA	0.21	Cu	483
PEEK	0.25	Al	204
PDMS	0.19	ZnO	60
PP	0.21	SiO_2_	32
PTFE	0.27	GO	5000
PS	0.19	CNT	3000
PVC	0.21	Diamond	2000

**Table 3:** Comparison of theoretical models [15,111,112,114]. Copyright 2025, Elsevier. Copyright 2021, Elsevier. Copyright 1962, American Chemical Society. Copyright 2025, Elsevier.

Model Type	Physical Mechanism	Applicable Scenarios
AMM/DMM	phonon volatility	low-temperature single-crystal interface
H-S/H	equivalent medium approximation	spherical/ellipsoidal packing
Foygel	transpiration network topology	high-aspect-ratio packing network
MD/FEA	atomic motion/continuum equations	complex interface microstructure

**Table 4:** Comparison of thermal conductivity properties of graphene-reinforced epoxy resin composites.

EP Matrix	Filler Additives	λ/(W·m^−1^·K^−1^)	Thermal Conductivity Enhancement/%	Refs.
Epoxy resin	19 wt%GO/EP	0.49	157	Zhu [138]
Epoxy resin (YDF-170)	15 vol%VA-GF/EP	0.96	465	Thieu [91]
Bisphenol-F-type EP (Epikote 862)	15 wt% NiCo-GNS/PVDF/EP	1.05	453	Jia [139]
Epoxy resin	5 wt%rGO-BTA@HMS/EP	1.239	596	Yang [140]
Bisphenol-A-type epoxy resin	MGHN/EP	1.5596	766	Luo [120]
Epoxy resin (E6002)	9 wt% PPD-rGO/EP	1.7	750	Lin [141]
Bisphenol-F epoxy (Epon 862)	3.98 wt%rGO-ERG/EP	1.96	931	Han [80]
Epoxy resin	2.5 wt%F-3DGA/EP	2.53	1388	Cui [83]
DGEBA-based epoxy resin (ARALDITE LY1564)	22 vol%S-BN/rGO/EP	3	1479	Hong [81]
Epoxy resin (E-51)	46 vol%TA@BN-rGO-CNT/EP	5.65	2873	Li [75]
Epoxy resin (LY1564)	5 wt%f-GnP/SiCnw/EP	6.2	3000	Wang [88]
Epoxy resin	9.10 wt%GO/EP	6.81	3683	Ma [62]
Bisphenol-F-type liquid epoxy resin (YD-170)	8.04 vol%PUF@GF/EP	9.68	5132	Mani [142]
Epoxy resin (waste)	33.9 vol%GNP/WEP/EP	10.1	4865	Kang [143]
Liquid epoxy resin	60 vol%3D-GO/EP	16.01	8326	Hu [144]
Epoxy resin (CY 230-1)	8 vol%Cu/10vol%CB/42vol%NFG/EP	17	8847	Mathew [145]
Epoxy resin (LH288)	4wt%GN/EP	40.6	21,268	Zambrzycki [146]
Epoxy resin	11.22 wt%GO	69.74	36,605	Ma [14]
Epoxy resin (NPEL-128)	5 wt%rGO/BaSO4/EP	165	86,742	Yung [147]
Bisphenol-A epoxy resin	23.3 vol% GO/CF/EP	262	137,794	Lu [61]

**Table 5:** Comparison of electronic packaging material properties [13,19,34,102,146]. Copyright 2024, Elsevier. Copyright 2025, Royal Society of Chemistry. Copyright 2024, Elsevier. Copyright 2022, Springer Nature. Copyright 2021, MDPI.

Material Type	λ/(W·m^−1^·K^−1^)	Density/(g·cm^−3^)	Processing Compatibility
EP	0.17–0.20	1.1–1.3	Excellent
Al_2_O_3_/EP	1.5–2.5	1.8–2.2	Moderate (with filler settling)
GO/EP	5–40.6	1.2–1.5	Good
Ceramic matrix composites	15–30	2.5–3.8	Poor (high brittleness)

**Table 6:** Comparison of thermal conductivity and electromagnetic shielding effectiveness of graphene-reinforced epoxy resin composites.

EP Matrix	Filler Additives	λ/(W·m^−1^·K^−1^)	EMI SE/dB	Refs.
Bisphenol-F epoxy (Epon 862)	3.98 wt%rGO-ERG/EP	1.96	45.9	Han [80]
Resin film	19 wt%GO/CFF	0.49	51.94	Zhu [138]
Bisphenol-F-type EP (Epikote 862)	15 wt%NiCo-GNS/EP	1.11	34.62	Jia [139]
Epoxy Acrylate Resin (Derakane-441)	6 wt% GSP/EA	2.13	45.93	Du [167]
Epoxy resin 6002	4.14 wt%GO/C/EP	1.19	35.23	Ba [69]
Bisphenol-F epoxy (Epon 862)	3.68 vol%VG-CNT/EP	2.23	46.9	Han [86]
Waste epoxy	33.9 vol%GNP/WEP/EP	10.1	106.3	Kang [143]
Epoxy resin (E-44)	10 wt%ZnO/EP	0.55	3.3	Leng [1]
Epoxy resin (JY-257)	20 vol%LMPA/ER	1.23	20	Zhang [169]
P-aminophenol epoxy resin	34.64 vol%Al_2_O_3_/Al_2_O_3_@Fe3O4/EP	1.83	10.6	Guo [170]
EP(JY-257)	35 wt%T-Fe_3_O_4_@CNTs/EP	1.59	45.86	Da [171]

## Data Availability

No new data were created or analyzed in this study. Data sharing is not applicable to this article.
